# Trends in Emergency Neurosurgical Workload: Evidence From a Tertiary Center

**DOI:** 10.7759/cureus.91543

**Published:** 2025-09-03

**Authors:** Hamath A Dorby, Edward J St. George

**Affiliations:** 1 Oral and Maxillofacial Surgery, Queen Elizabeth University Hospital, Glasgow, GBR; 2 Neurosurgery, Queen Elizabeth University Hospital, Glasgow, GBR

**Keywords:** chronic subdural haematoma, neurosurgical centre, neurosurgical emergency referrals, resource allocation, retrospective audit, spinal and cranial trauma

## Abstract

Background

Emergency neurosurgical referrals are a leading driver of on-call workload and unplanned admissions. Tracking their volume and case-mix supports safe staffing, imaging capacity, and bed planning across regional networks. The study included all emergency referrals made to the department between 2020 and 2022.

Methodology

Patient data were individually extracted from a prospectively maintained local database. The initial step involved a de-duplication process, ensuring the dataset represented unique patient referrals for each year under consideration. The subsequent phase involved a nuanced categorization strategy to distinctly identify new case referrals against follow-ups. An advanced synonym recognition approach was adopted to include a range of terms and related clinical conditions, ensuring robust and inclusive data categorization. The data were analyzed in R Studio using negative binomial regression (monthly call counts, adjusted for seasonality), quasi-Poisson regression (sensitivity analysis of annual totals), the Kruskal-Wallis test (nonparametric comparison of monthly counts across years), Kendall’s τ trend test (monotonic trend in annual counts), and logistic regression (odds of out-of-hours (OOH) vs. in-hours (InH) calls, adjusted for month).

Results

The data revealed a progressive increase in calls to the neurosurgical registrar, from 5,435 in 2016 to 9,567 in 2021 and 9,279 in 2022 (quasi-Poisson incidence rate ratio (IRR), 1.08 per year; 95% confidence interval (CI), 1.06-1.11; *P* < 0.001). After seasonality adjustment, monthly referrals increased by 30% in 2021 (IRR 1.30, 95% CI 1.17-1.46) and 22% in 2022 (IRR 1.22, 95% CI 1.11-1.34) vs. 2020. Transfers climbed from 3,100/18,128 (17%) in 2016-2018 to 6,939/26,202 (27%) in 2020-2022. The proportion of calls logged OOH was unchanged (odds ratio (OR), 0.97 in 2021; 0.91 in 2022). The median transfer age was 56 years (interquartile range (IQR), 45-68), and 46% of patients were male. Cauda equina syndrome and chronic subdural hematoma accounted for 2,428 transfers (26%) in 2022, each representing an increase of 1,365 cases (78%) compared with 2020.

Conclusions

We observed year-on-year growth in emergency referrals, with a concurrent rise in transfers. While crude trends cannot distinguish true incidence from behavioral change, the data are consistent with a lowered operational threshold for both referral and transfer. Service planning should therefore prioritize rota resilience, ring-fenced urgent imaging, optimization of bed capacity, and clearer referral pathways; routine monitoring should be maintained to detect emerging risks to access and outcomes.

## Introduction

The Glasgow Neurosurgical Department is based at the Institute of Neurological Sciences (INS), Queen Elizabeth University Hospital, and is Scotland’s largest neuroscience unit, serving approximately 2.6 million people across NHS Greater Glasgow and Clyde (NHSGGC), Lanarkshire, Ayrshire and Arran, the Western Isles, and parts of Highland [1]. Emergency referrals are received via a 24/7 on-call hub. Pathway details, including spinal injury and thoracolumbar fracture routing, vary by geography but were unchanged during the study period [1]. Growing referral volumes and transfer activity place pressure on staffing, urgent imaging, and bed availability. Contemporary system factors, COVID-19 disruption, population aging with increased anticoagulant exposure, and medico-legal caution regarding suspected cauda equina, may be lowering thresholds for referral and transfer.

The aim of this study was to quantify referral trends to the INS neurosurgical unit(2020-2022) and evaluate out-of-hours (OOHs) activity, transfers, demographics, and key pathology subgroups using seasonality-adjusted regression models, building on Spencer et al. [2]

## Materials and methods

Study design

A retrospective audit of adult emergency referrals to the INS for the years 2020 to 2022 was undertaken. Data were obtained from a prospectively maintained, locally held Excel database. Details of each call made to the INS, along with any follow-up communications, were recorded in the dataset, and domains including date, time of referral, parent neurosurgical consultant, Community Health Index (CHI) number, referring hospital/ward, clinical history, and management plan were interrogated.

Inclusion criteria

Adult patients (≥16 years) referred via the neurosurgical on-call system between January 1, 2020, and December 31, 2022, with a complete date/time stamp, a valid Community Health Index (CHI) number, a documented primary pathology, and representing the patient’s first neurosurgical contact within that calendar year.

Exclusion criteria

Patients younger than 16 years, duplicate records (same CHI and date), entries lacking any of the essential fields listed above, and referrals that were elective or otherwise non-emergency in nature.

Data collection

**Figure 1:**
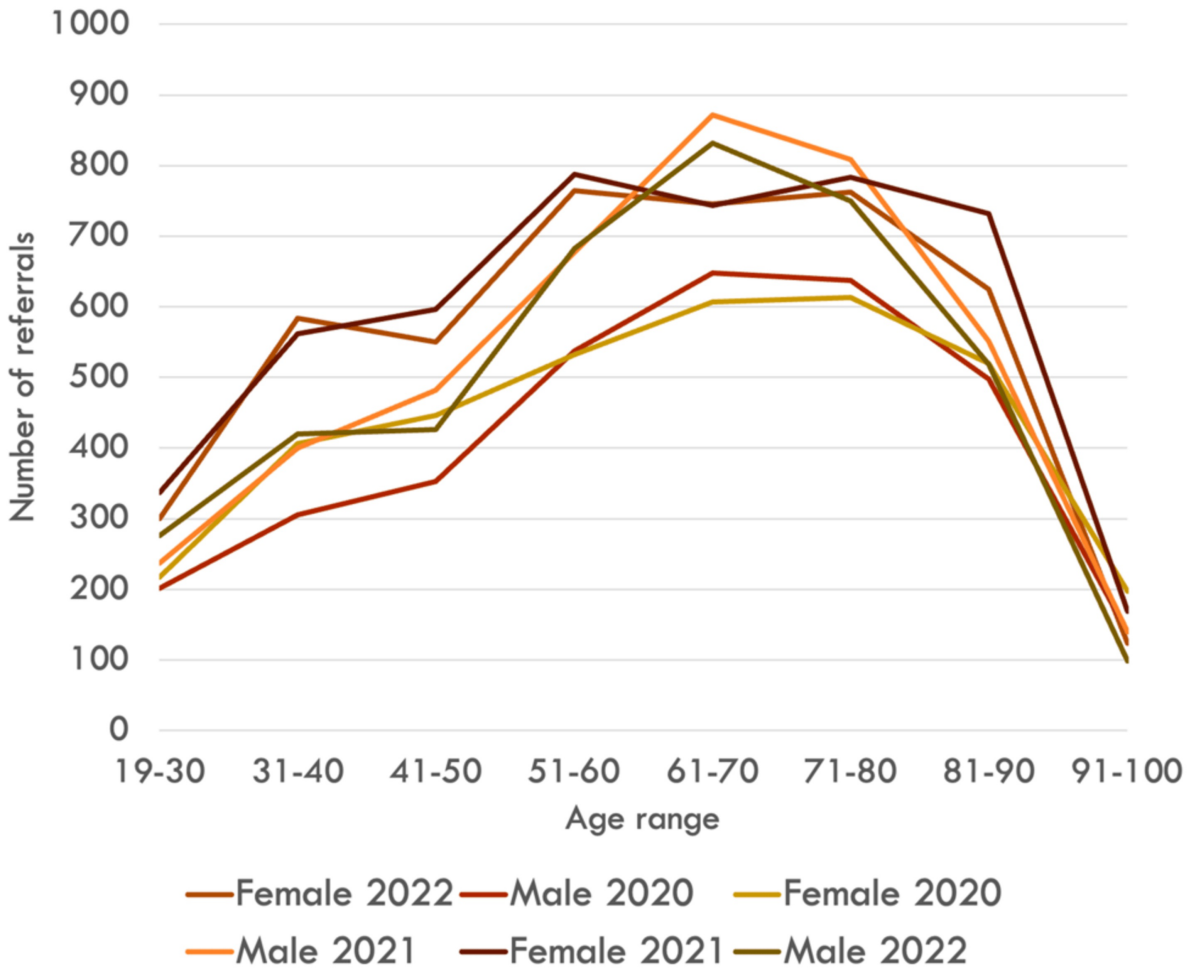
Demographic data and sex distribution. Age distribution of referrals. Sex distribution overall and by age band. Notable finding: women aged 19-30 years showed a sharp rise from 2020 to 2021, with a partial reversal in 2022, whereas men increased steadily.

For demographic analysis, the first six digits of the CHI number were used, which is a unique patient tracking number comprising a patient’s date of birth and a randomly generated four-digit number where the penultimate digit (odd or even) is determined by patient gender. The total number of calls was extracted directly from the dataset. Cases that were transferred were identified by scrutinizing the presenting history and management plan documented for each patient. Search terms were used to identify the index pathologies depicted in Figure 1. These data were further filtered manually. For example, 40 search terms were used to identify cases of traumatic brain injury (TBI), which included "TBI", "Traumatic Brain Injury", "Brain Trauma", "Head Injury", "Brain Injury", and "Concussion" (Table 1). Once identified, the cases were further examined to determine which patients actually presented with a TBI rather than merely having a history of the condition.

**Table 1:** Term-family dictionaries used to screen referral free text for each pathology domain, the exclusion rules applied during manual adjudication (to avoid false positives, e.g., “rule-out” or “old/remote” events), and worked examples of classification decisions. Term-family keyword dictionaries and exclusion rules by pathology domain. Purpose: To support the reproducibility of case identification when exact historical string lists are unavailable. Columns: Domain = pathology category used in reporting; Inclusion term families (examples) = common phrases used to identify candidates (illustrative, not exhaustive); Exclusion rules (examples) = phrases/contexts triggering manual exclusion; Notes/adjudication examples = brief examples of how ambiguous cases were handled. Notes: Term families reflect clinically meaningful groupings (e.g., “subdural/SDH/CSDH”) rather than exact strings. Manual verification applied these rules to avoid false positives (e.g., “rule-out,” “history of,” follow-up only). CES, cauda equina syndrome; CSDH, chronic subdural hematoma; SDH, subdural hematoma; SOL, space-occupying lesion; LVO, large-vessel occlusion

Domain	Inclusion term families (examples)	Exclusion rules (examples)	Notes/adjudication examples
CES	cauda equina; CES; saddle anesthesia; urinary retention; perineal numbness	“rule out/?”, “history of/resolved”, normal MRI	Count only new/suspected acute CES; exclude past CES without new neuro signs
CSDH	subdural; SDH; CSDH; “chronic subdural”	“old/remote SDH”, follow-up only	Include when a new symptomatic CSDH or a new referral for management occurs
SOL	space-occupying lesion; SOL; brain mass; tumour/tumor; mass effect	metastatic work-up only” w/ no acute neurosurg question	Label SOL when acute mass effect or neurosurgical advice is sought
Stroke	ischemic stroke/ischemic; infarct; thrombectomy; large vessel occlusion/LVO; MCA occlusion	TIA; stroke mimic	Stroke domain when acute neurovascular referral or thrombectomy query
Infection	abscess; empyema; ventriculitis; encephalitis	colonized/contaminant; resolved	Include when an active intracranial infection is suspected/confirmed

Statistical analysis

All analyses were performed in R 4.5.0 (R Foundation for Statistical Computing) using the packages sandwich, lmtest, broom, tidyverse, and ggplot2. Monthly call counts (January 2020 to December 2022) were the primary unit of observation. Temporal changes in volume were examined using a log-linear quasi-Poisson regression that included calendar month as a 12-level factor to control for seasonality and employed HC0 robust standard errors to accommodate over-dispersion (Pearson χ²/df ≈ 18). Effect sizes were reported as incidence rate ratios (IRR) with 95% confidence intervals. A negative binomial model and the Kruskal-Wallis test provided sensitivity checks and yielded concordant results.

To place these findings in a longer-term context, annual totals for 2016, 2018, 2019, and the aggregated monthly counts for 2020-2022 were fitted to a quasi-Poisson model with centered calendar year as a continuous predictor, yielding an average year-on-year IRR (overdispersion: Pearson χ²/df ≈ 63). Additional robustness was assessed using a negative binomial fit and a Kendall rank-correlation test.

Finally, the monthly percentage of calls made OOH was analyzed using a seasonality-adjusted quasi-binomial GLM (cbind(OOH, InH) ~ month + year, reference = 2020). ORs with 95% CIs were obtained by exponentiating coefficients, and dispersion was acceptable (Pearson χ²/df ≈ 0.81). All *P*-values were two-sided, and figures were generated using ggplot2.

## Results

**Figure 2:**
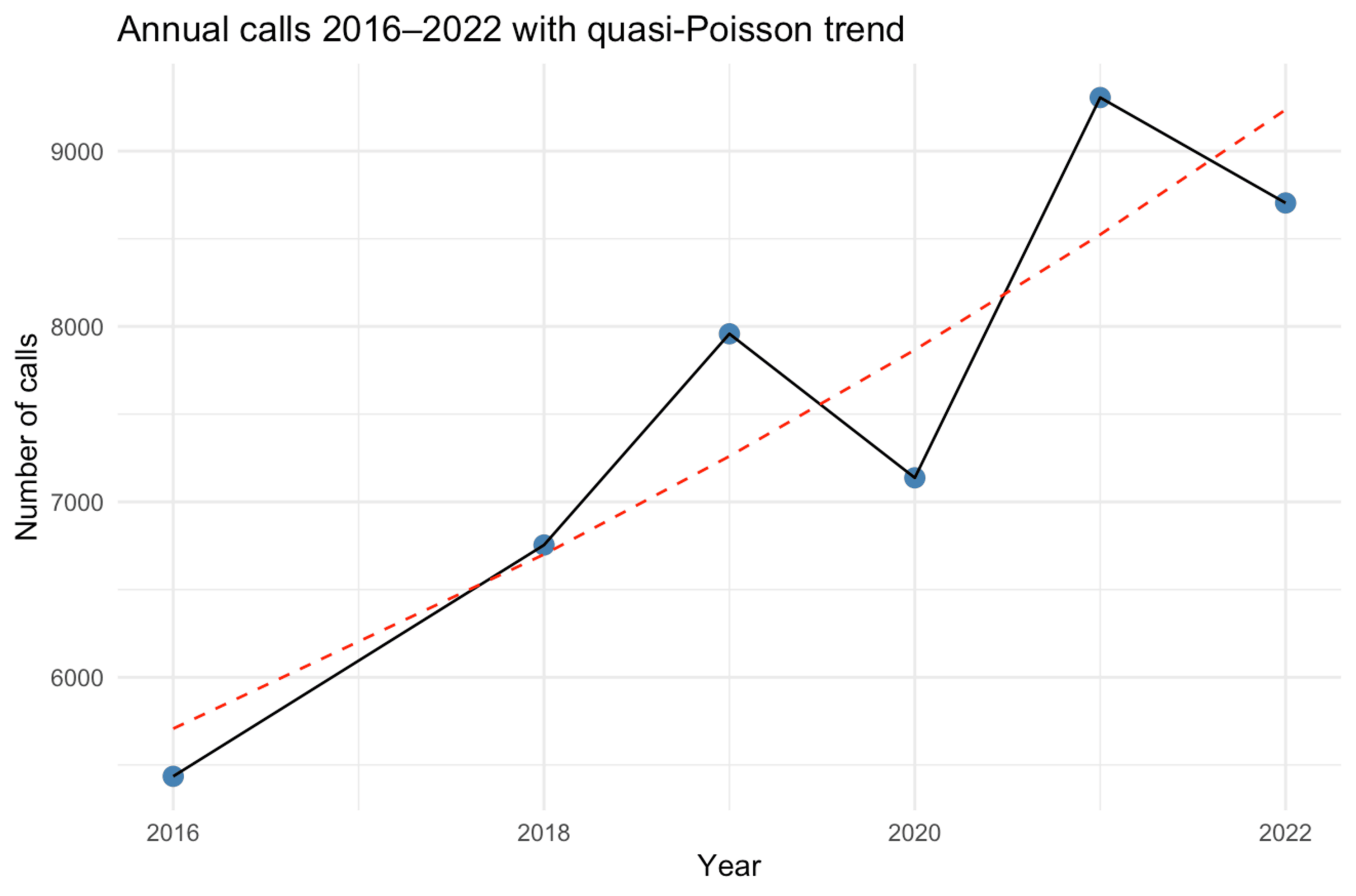
Number of calls each year. Annual emergency neurosurgical referrals, 2016-2022. Bars show yearly totals of unique referrals to the adult neurosurgical on-call service; 2017 logs were unavailable. The dashed annotation reports the average year-on-year change from a quasi-Poisson model of annual totals (2016, 2018-2022) with centered calendar year as a continuous predictor. A negative binomial fit and Kendall’s τ provided concordant sensitivity checks. IRR, incidence rate ratio

From 2020 to 2022, there was a notable increase in the number of calls, with figures increasing from 7,356 in 2020 to 9,567 in 2021, with a slight decrease to 9,279 in 2022, as shown in Figure 2.

Call volume also increased on a longer time scale: a quasi-Poisson regression of annual totals estimated an 8% average rise per year from 2016 to 2022 (IRR = 1.08, 95% CI 1.06-1.11; *P* < 0.001) (Figure 2). The negative binomial sensitivity model gave a similar point estimate but wider uncertainty (IRR = 1.09, 95% CI 0.72-1.63) owing to only six annual observations, while a Kendall trend test pointed in the same direction (τ = 0.73; *P *= 0.056). Collectively, the analyses document a steady rise in referrals from 5,435 in 2016 to 9,567 in 2021 and 9,279 in 2022, despite missing data for 2017.

**Figure 3:**
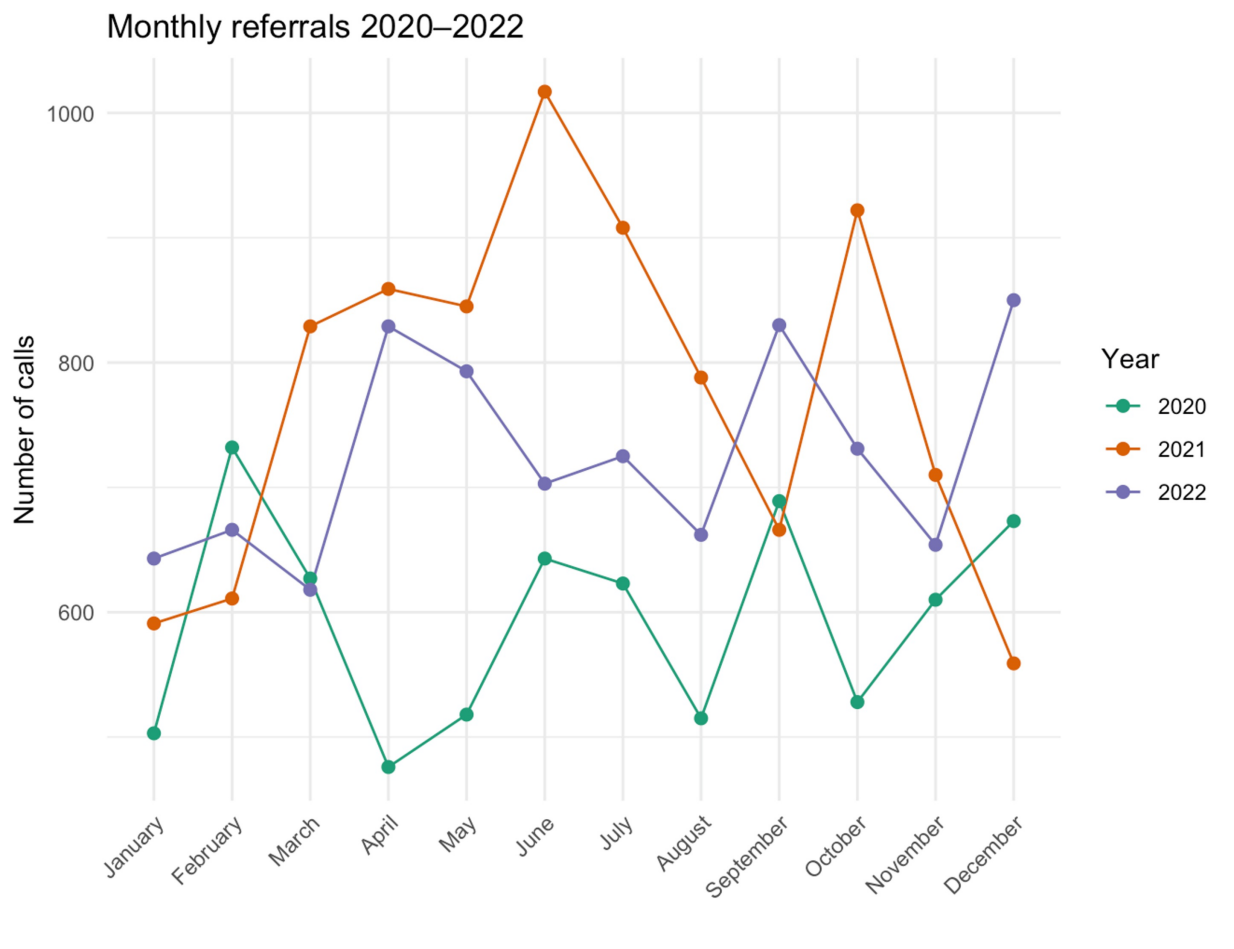
Number of calls per month, by year. Monthly referrals by year, 2020-2022. Lines show monthly counts; shading indicates 95% CIs from a negative binomial model with calendar-month fixed effects (seasonality). Labels show seasonality-adjusted IRRs for 2021 and 2022 versus 2020. Population: unique emergency referrals to the adult neurosurgical on-call service. CI, confidence interval

After seasonality adjustment, monthly referrals rose by 30% in 2021 (IRR = 1.30, 95% CI 1.17-1.46; *P* < 0.001) and 22% in 2022 (IRR = 1.22, 95% CI 1.11-1.34; *P* < 0.001) relative to 2020; a negative-binomial model and a Kruskal-Wallis test confirmed the upward trend (Figure 3).

**Figure 4:**
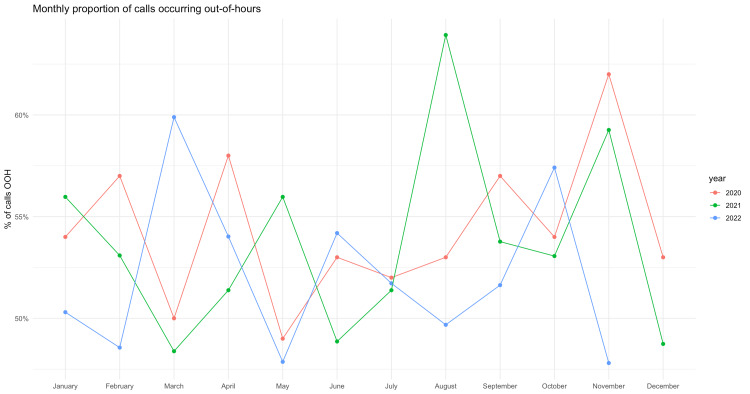
Percentage of calls occurring out-of-hours (OOH). OOH referrals over time. Monthly proportion of OOH referrals. Adjusted ORs for OOH referral by year from a seasonality-adjusted quasi-binomial model. Points represent estimates; bars represent 95% CIs. OR, odds ratio; CI, confidence interval

For the OOH metric, model fit showed mild underdispersion (Pearson dispersion = 0.81). After month adjustment, the odds of a call occurring OOH were essentially unchanged in 2021 (OR = 0.97, 95% CI 0.84-1.12) and only marginally, yet non-significantly, lower in 2022 (OR = 0.91, 95% CI 0.78-1.05), indicating no secular shift in the proportion of calls handled OOH (Figure 4).

Referrals for women aged 19-30 years showed a relative increase of 55.3% from 2020 to 2021, followed by a relative reduction of 11% from 2021 to 2022. In contrast, male referrals in this age group increased steadily over the same period. Females showed marked fluctuations in referral numbers compared to their male counterparts, particularly in the 19-30-year age group, as depicted in Figure 1. These sex-age patterns were pre-specified and are examined in the Discussion for potential drivers (case-mix, anticoagulation exposure, and referral behavior).

**Figure 5:**
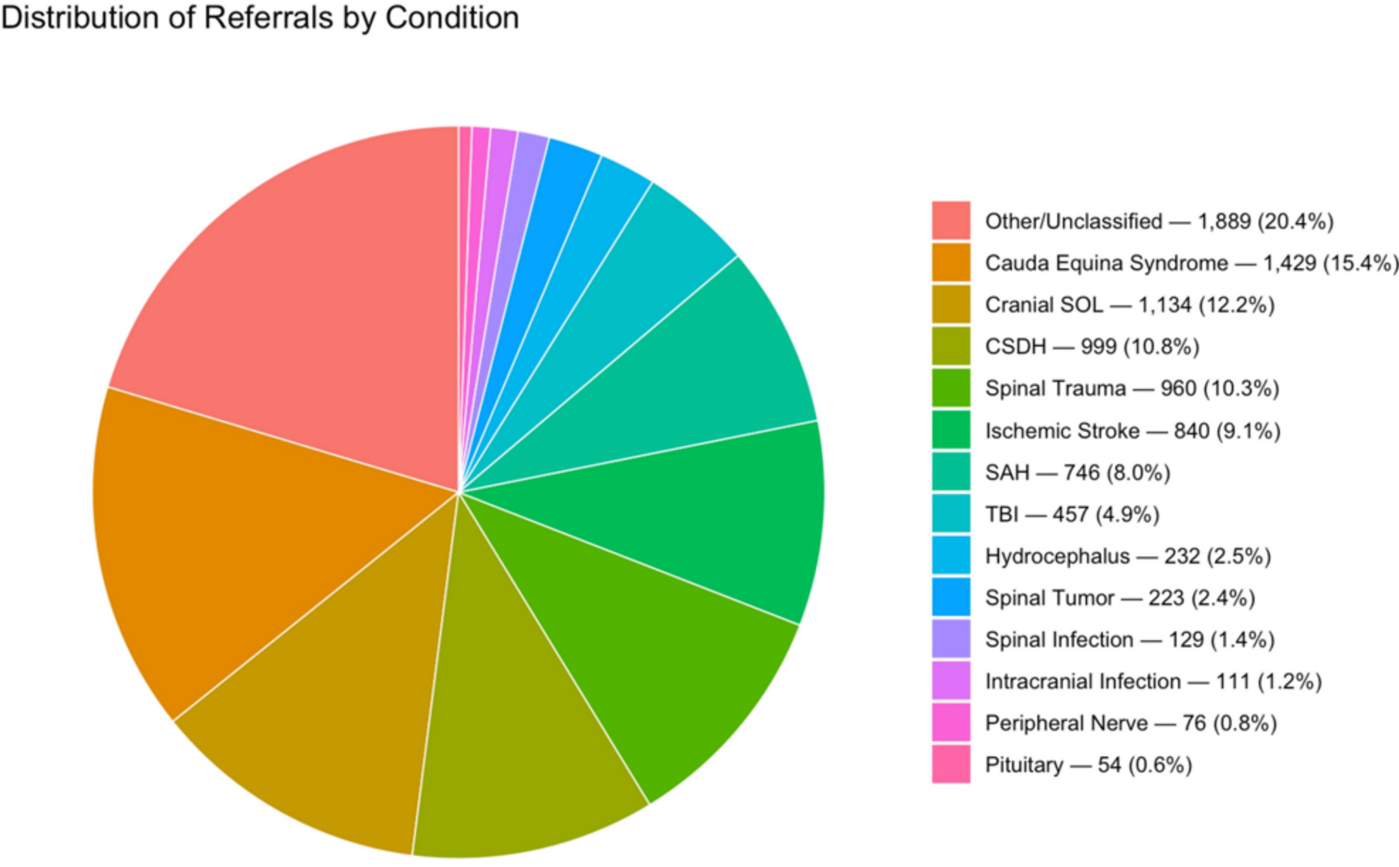
Distribution of cases in 2022 (n, %). Pathology mix in 2022 (*n*, %). Top categories include CES and CSDH, both of which increased compared with 2020. Case identification was performed using keyword searches with manual verification. SOL, space-occupying lesion; CES, cauda equina syndrome; CSDH, chronic subdural hematoma; TBI, traumatic brain injury; SAH, subarachnoid hemorrhage

An increase was observed across the majority of pathological categories, particularly CES and chronic subdural hematoma (CSDH). Between 2020 and 2022, CES cases increased from 817 (reference, 2020) to 1,354 (65.7% vs. 2020) in 2021, and 1,429 (74.9% vs. 2020; 5.5% vs. 2021) in 2022. CSDH cases mirrored this trend. Cranial space-occupying lesion (SOL) cases rose from 725 (reference, 2020) to 936 (29.1% vs. 2020) in 2021, and 1,134 (56.4% vs. 2020; 21.2% vs. 2021) in 2022. Intracranial infections increased from 68 (reference, 2020) to 89 (30.9% vs. 2020) in 2021, and 111 (63.2% vs. 2020; 24.7% vs. 2021) in 2022. Cases of ischemic stroke increased from 503 (reference, 2020) to 742 (47.5% vs. 2020) in 2021 and 840 (67.0% vs. 2020; 13.2% vs. 2021) in 2022 (Figure 5).

## Discussion

The articles by Spencer et al. and Mukerji et al. reported significant increases in neurosurgical referrals over their respective study periods [2,3]. Both groups advocated expanding specialist staffing and upgrading infrastructure to meet the growing workload, stressing the need for resource optimization and service innovation. Medicolegal considerations, particularly in conditions such as CES, were suggested as contributing factors. Defensive medical practices, influenced by the medico-legal environment, lower referral thresholds, and contribute to the increasing incidence of CES referrals [2-5,6-9]. Improving access to OOH imaging, especially MRI, for suspected CES and updating referral guidelines to streamline pathways align with this service-level response [5,9]. We observed persistent sex- and age-related differences in referral and transfer, likely reflecting case mix, anticoagulation/frailty, and referral behavior. These patterns warrant subgroup-targeted pathways, point-of-referral decision support, and protected OOH imaging to reduce avoidable transfers.

Internationally, similar pressures have been reported: population-based and multicenter studies describe rising burdens of CSDH in aging populations with wider antithrombotic exposure, and many systems have centralized neurosurgical care, resulting in associated increases in transfers. These parallels suggest that the UK trends we report are not unique, although absolute rates and service configurations vary across countries [10,11].

A more critical view of causation is warranted. Our observational data cannot disentangle growth in true neurosurgical pathology from behavioral and organizational effects: lowered thresholds for referral/transfer (including medicolegal caution around CES), pandemic-related changes to access and workflow (e.g., imaging availability, remote triage, theater prioritization), and further centralization of specialist services could all increase referral volumes without a commensurate rise in incidence. Accordingly, our findings should be interpreted as the net product of incidence, pathway design, and clinician behavior rather than pure disease trends.

The incidence of CSDH has increased due to an aging population, greater use of direct oral anticoagulants (DOACs), and a lower threshold for intervention [12-14]. Resource allocation must, therefore, be aligned to such change. Investment in equipment is also crucial, but not sufficient. Innovation to increase capacity and improve resource efficiency for sustainable service improvement, along with adapting resources and practices to cope with increasing demand, is necessary. The role of artificial intelligence (AI) should be considered as a hypothetical future research direction rather than an immediate solution: potential applications include supporting referral triage and imaging prioritization, but these require rigorous prospective validation, bias/inequity assessment, integration into clinical workflow, and clear information-governance safeguards before adoption [15]. The impact of the COVID-19 pandemic is reflected in this study [16], a factor that did not influence previous authors’ data. Despite meticulous planning, unforeseen events such as the COVID-19 pandemic can disrupt even the most carefully crafted strategies. This underscores the importance of building efficient and resilient systems, driven by data-informed decision-making, to navigate uncertainties and sustain progress. 

Limitations 

This single-center retrospective audit used call-log data with keyword-based case identification, so misclassification and missed cases are possible despite manual checks. Classification depended on clinician coders; coder bias and drift are possible, and interrater agreement was not assessed. Missing 2017 data and the absence of linked patient-level outcomes restrict conclusions to workload patterns rather than patient safety or clinical outcomes (e.g., time to imaging or definitive management, complications, length of stay, and re-presentations). Pandemic-related service changes and other unmeasured confounders may have distorted trends. Transfer proportions reflect available referral denominators and may not capture all inter-hospital movements. Demographic sub-analyses relied on routinely recorded fields rather than full notes, introducing information bias. Accordingly, findings may not generalize beyond our regional service and should be validated prospectively in multi-center studies, ideally with case-note verification, inter-rater reliability assessment, and linkage to outcomes. We did not implement or test any AI systems; statements about AI are therefore speculative and require validation, as outlined above. Exact string-level keyword logs were not retained contemporaneously; therefore, case identification relied on a single-coder term-family search with manual adjudication, and no interrater statistic is available. To support replication, we provide worked examples and exclusion rules in Table 1. The operational call log contains patient-level information and is not publicly shareable.

## Conclusions

Emergency neurosurgical referral demand increased across the study period, signaling sustained structural pressure on services. Although causality cannot be assigned, plausible system-level drivers include lower referral thresholds, defensive practice, and service centralization. The result is rising demand on a finite workforce and bed base, with attendant risks to timeliness, staff well-being, and, if unaddressed, patient safety.

Commissioners, health boards, and service leads should coordinate workforce planning (rota capacity and senior decision-maker availability), protect urgent imaging and bed/theater capacity (ring-fenced access), and streamline referral processes (standardized criteria with point-of-referral decision support). As a single-center audit of call-log data without outcomes linkage, these findings warrant prospective validation with outcome capture and ongoing surveillance. Near-real-time monitoring should be used as an early-warning system to anticipate bottlenecks and trigger timely mitigations.
